# f-Element Zintl Chemistry: Actinide-Mediated Dehydrocoupling of H_2_Sb^1–^ Affords the Trithorium and Triuranium Undeca-Antimontriide Zintl Clusters [{An(Tren^TIPS^)}_3_(μ_3_-Sb_11_)] (An = Th, U; Tren^TIPS^ = {N(CH_2_CH_2_NSi^i^Pr_3_)_3_}^3–^)

**DOI:** 10.1021/acs.inorgchem.4c00923

**Published:** 2024-05-20

**Authors:** Jingzhen Du, Kevin Dollberg, John A. Seed, Ashley J. Wooles, Carsten von Hänisch, Stephen T. Liddle

**Affiliations:** † Department of Chemistry and Centre for Radiochemistry Research, The University of Manchester, Oxford Road, Manchester, M13 9PL, United Kingdom; ‡ Fachbereich Chemie, Philipps-Universität Marburg, Hans-Meerwein-Straße 4, 35043 Marburg, Germany

## Abstract

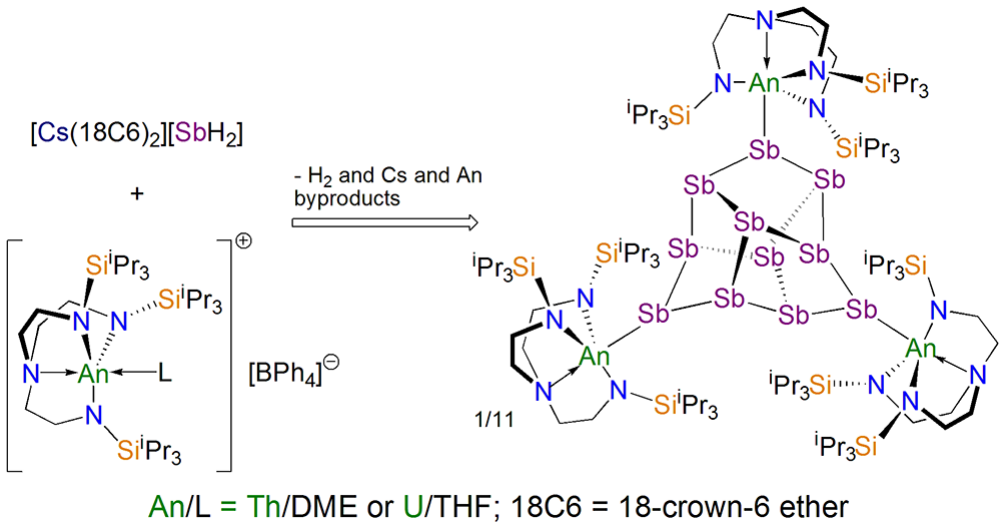

Reaction of the cesium antimonide complex [Cs(18C6)_2_][SbH_2_] (**1**, 18C6 = 18-crown-6 ether) with the triamidoamine actinide separated ion pairs [An(Tren^TIPS^)(L)][BPh_4_] (Tren^TIPS^ = {N(CH_2_CH_2_NSi^i^Pr_3_)_3_}^3–^; An/L = Th/DME (**2Th**); U/THF (**2U**)) affords the triactinide undeca-antimontriide Zintl clusters [{An(Tren^TIPS^)}_3_(μ_3_-Sb_11_)] (An = Th (**3Th**), U (**3U**)) by dehydrocoupling. Clusters **3Th** and **3U** provide two new examples of the Sb_11_^3–^ Zintl trianion and are unprecedented examples of molecular Sb_11_^3–^ being coordinated to anything since all previous reports featured isolated Sb_11_^3–^ Zintl trianions in separated ion quadruple formulations with noncoordinating cations. Quantum chemical calculations describe dominant ionic An–Sb interactions in **3Th** and **3U**, though the data suggest that the latter exhibits slightly more covalent An–Sb linkages than the former. Complexes **3Th** and **3U** have been characterized by single crystal X-ray diffraction, NMR, IR, and UV/vis/NIR spectroscopies, elemental analysis, and quantum chemical calculations.

## Abstract 2

Reaction of [Cs(18C6)_2_][SbH_2_] (**1**, 18C6 = 18-crown-6 ether) with the [An(Tren^TIPS^)(L)][BPh_4_] (Tren^TIPS^ = {N(CH_2_CH_2_NSi^i^Pr_3_)_3_}^3−^; An/L = Th/DME (**2Th**); U/THF (**2U**)) affords the triactinide undeca-antimontriide Zintl clusters [{An(Tren^TIPS^)}_3_(μ_3_-Sb_11_)] (An = Th (**3Th**), U (**3U**)). These clusters provide unprecedented examples of the Sb_11_^3−^ trianion being coordinated to anything in a molecular context, with all prior examples involving separated ion quadruples. Quantum chemical calculations suggest that the U−Sb bonds are marginally more covalent than the Th−Sb linkages.

## Introduction

Although the chemistry of actinide (An)–nitrogen bonds can be considered to be fairly mature,^1−10^ heavier group 15 analogs are less well developed.^11^ Indeed, the numbers of An–P, −As, −Sb, and −Bi bonds falls away rapidly as group 15 is descended, and An–Sb and −Bi derivatives are particularly rare.^12−17^ To address this situation we have been exploring the chemistry of An–Sb bonds.^13,16,17^ After initial investigations involving (Me_3_Si)_2_Sb^1–^ and (Me_3_Si)_2_C(H)Sb(H)^1–^,^13,16^ the latter of which enabled access to a new molecular Zintl (Sb_3_Li_4_Sb_3_)^2–^ dianion,^16^ we turned our attention^17^ to the parent antimonide H_2_Sb^1–^ to examine whether other An–Sb linkages and Zintl clusters might be accessible since up to Sb_8_^4–^ has been stabilized by lanthanides.^18^ Specifically, we found that the potassium derivative, the contact ion pair [K(18C6)(THF)SbH_2_] (18C6 = 18-crown-6 ether),^19^ reacted with a range of triamidoamine-thorium cyclometallate and separated ion pair complexes to afford access to discrete bridging stibido Th=Sb=Th, stibinidiide Th–Sb(H)–Th, stibinidiide Th=Sb(H)K(18C6), stibinidene Th=Sb(H), distibene Th(μ–η^2^:η^2^–Sb_2_)Th, and tetrameric stibido (Th≡SbK_2_)_4_ structural motifs.^17^ The potassium antimonide is part of a series of alkali metal derivatives developed by some of us,^19^ and so our attention moved to investigating the reactivity of the other alkali metal reagents. In particular, the potassium antimonide is a contact ion pair, so we were interested to assess how the cesium analog, [Cs(18C6)_2_][SbH_2_] (**1**),^19^ would react, since it is a separated ion pair, and this would present the opportunity to probe the reactivity of the naked parent antimonide H_2_Sb^1–^ anion free of coordinated metal ion effects.

Here, we report on reactions of **1** with [An(Tren^TIPS^)(L)][BPh_4_] (Tren^TIPS^ = {N(CH_2_CH_2_NSi^i^Pr_3_)_3_}^3–^; An/L = Th/DME (**2Th**); U/THF (**2U**)),^20,21^ which both produce the trithorium and triuranium undeca-antimontriide Zintl clusters [{An(Tren^TIPS^)}_3_(μ_3_-Sb_11_)] (An = Th (**3Th**), U (**3U**)). These clusters evidently form via dehydrocoupling reactions, emphasizing the underlying drive of heavier group 15 elements toward catenation and cluster formation, and they provide two new, structurally authenticated, examples of the Sb_11_^3–^ Zintl trianion, which remains rare with only seven structurally authenticated examples reported and none of them involve actinide ions.^22−28^ Furthermore, **3An** constitute unprecedented examples of molecular Sb_11_^3–^ being coordinated to anything, since the few structurally authenticated examples of the Sb_11_^3–^ Zintl trianion in the literature all feature isolated Sb_11_^3–^ Zintl trianions in separated ion quadruple formulations involving noncoordinating cations.^22−28^ The title complexes hence bring a new formulation of a rare Zintl cluster to the enormous field of Zintl chemistry.^29−31^

## Results and Discussion

### Synthesis

**Scheme 1:**
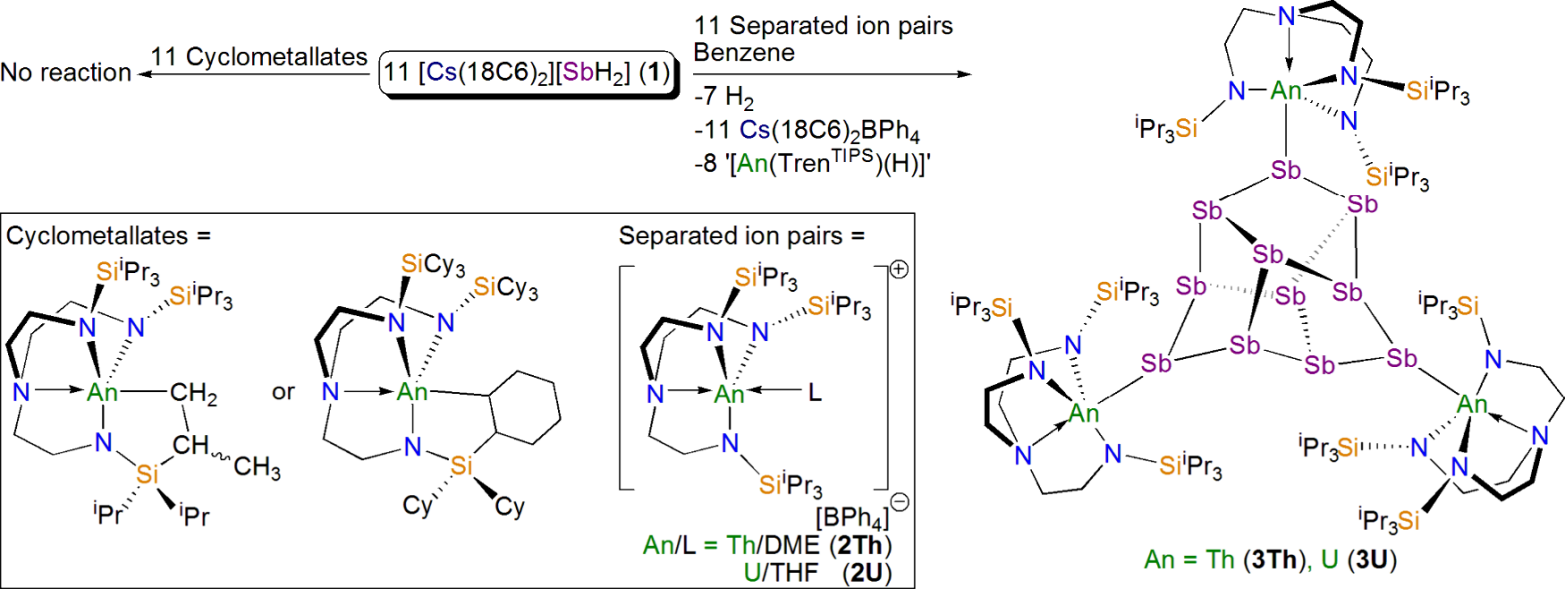
Non-Reactions of **1** with Cyclometallate Complexes and Synthesis of **3Th** and **3U** from **1** and **2An** The eight equiv of [An(Tren^TIPS^)(H)] by-products could in principle decompose to eight equiv of the cyclometallate complexes [An{N(CH_2_CH_2_NSi^i^Pr_3_)_2_(CH_2_CH_2_NSi^i^Pr_2_CHMeCH_2_)}], liberating another eight equiv of H_2_. This is the formal, balanced equation, noting that for **3U** significant quantities of [U(Tren^TIPS^)] by-product are also isolated implying uranium reduction in addition to, or instead of, the principal salt elimination/dehydrocoupling reaction.

Under the same conditions as our previous work with [K(18C6)(THF)SbH_2_]^17^ and that which furnishes the title complexes (see below), we initially attempted reactions of **1** with the triamidoamine cyclometallate complexes [An{N(CH_2_CH_2_NSi^i^Pr_3_)_2_(CH_2_CH_2_NSi^i^Pr_2_CHMeCH_2_)}] and [An{N(CH_2_CH_2_NSiCy_3_)_2_(CH_2_CH_2_NSiCy_2_[CHCH_2_CH_2_CH_2_CH_2_CH])}] (An = Th, U; Cy = cyclohexyl).^32,33^ However, in all cases no reactions occurred at room temperature (Scheme 1), and elevated temperatures induced decomposition. For the thorium congeners this directly contrasts to our previous report^17^ detailing the reactions of those thorium reagents with [K(18C6)(THF)SbH_2_],^19^ which afforded bridging stibido Th=Sb=Th and stibinidiide Th=Sb(H)K(18C6) linkages that could each be converted to stibinidiide Th–Sb(H)–Th and terminal stibinidene Th=Sb(H) motifs, respectively.^17^ We suggest that the potassium antimonide is reactive because through polarization effects the coordinated K-cation weakens the Sb–H bonds enough for protonolysis with the Th–C cyclometalate linkages to occur. However, since **1** is a separated ion pair with no bond polarization effects from the Cs cation, the Sb–H bonds are not activated enough for protonolysis reactions with the cyclometalated complexes. We therefore turned our attention to salt elimination reactions, noting that in our prior study we found that **2Th** reacts with the potassium antimonide reagent to afford a distibene Th(μ-η^2^:η^2^-Sb_2_)Th motif which can subsequently be reductively cleaved into a tetrameric stibido (Th≡SbK_2_)_4_ structure.^17^ Thus, in separate reactions with the exclusion of light we treated **2Th** and **2U** with **1** (Scheme 1), and after workup and recrystallization, we isolated [{An(Tren^TIPS^)}_3_(μ_3_-Sb_11_)] (An = Th (**3Th**), U (**3U**)) as black and red crystalline materials in 34 and <5% yield (by Sb-content), respectively.

During the reactions that afford **3Th** and **3U**, gas-evolution was observed for both, but substantially more was noted for the former than the latter and the isolated yield for **3Th** is greater than for **3U**. As shown in Scheme 1, in principle at least 7 equiv of H_2_ should be eliminated, which would be consistent with our experimental observations, so while the observation of gas-evolution is qualitative in nature, it is logical for it to be inextricably linked to the reactions yields since **3Th** and **3U** evidently form by salt elimination then dehydrocoupling mechanisms. Furthermore, formally eight equiv of [An(Tren^TIPS^)(H)] will form to provide mass balance, though these complexes could decompose to produce [An{N(CH_2_CH_2_NSi^i^Pr_3_)_2_(CH_2_CH_2_NSi^i^Pr_2_CHMeCH_2_)}] and H_2_ (a further eight equiv of each). We note that a significant quantity of trivalent [U(Tren^TIPS^)] is formed in the preparation of **3U**, before the hot extraction step, which when taken together with the low yield of **3U** implies redox at the expense of salt elimination/dehydrocoupling. Given that thorium(IV) is much more robust than uranium(IV) in terms of redox stability, we suggest that the significantly lower yield of **3U** compared to **3Th** reflects deleterious redox processes occurring for uranium that do not occur for thorium (for ^1^H NMR spectra of crude reaction mixtures see Figures S5 and S8).

Notwithstanding the above points about redox aspects of the formation of **3Th** and **3U**, it seems likely that the initial step of the reactions between **1** and **2Th**/**3U** is the formation of [An(Tren^TIPS^)(SbH_2_)], which then undergoes a sequence of dehydrocoupling reactions to eventually form **3Th** and **3U**. Indeed, in our prior report,^17^ [K(18C6)(THF)SbH_2_] was found to react with **2Th** forming [{Th(Tren^TIPS^}_2_(μ-η^2^:η^2^-Sb_2_)], again by dehydrocoupling, where [An(Tren^TIPS^)(SbH_2_)] is a logical intermediate. It would thus seem that the An–SbH_2_ linkage is highly reactive, but the reaction was not amenable to mechanistic study given its nature, and when local excess H_2_Sb^1–^ is available rather than forming Sb_2_^2–^ the larger Sb_11_^3–^ forms.

### Solid-State Structures

**Figure 1:**
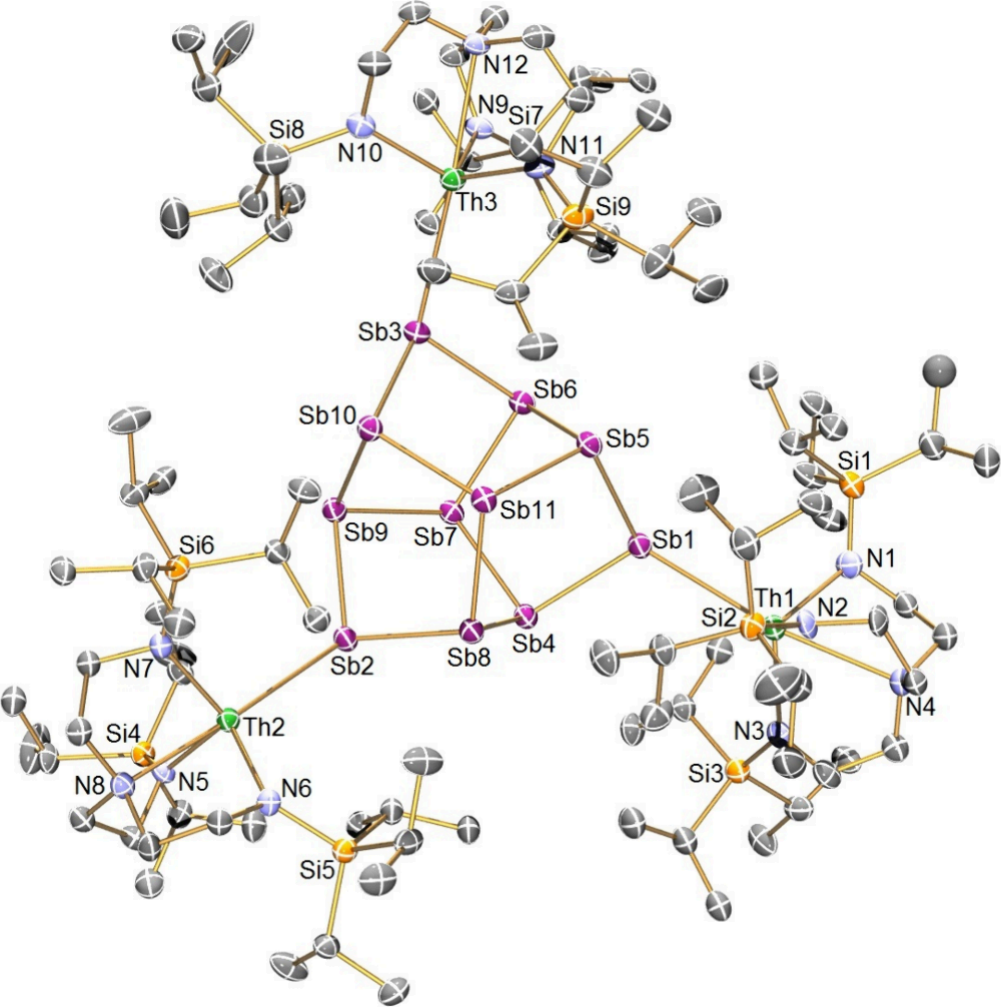
Molecular structure of **3Th** at 100 K with displacement ellipsoids set at 40%. Hydrogen atoms, disordered components, and lattice solvent molecules are omitted for the sake of clarity. The structure of **3U** is very similar.

The solid-state structures of **3Th** and **3U** were determined by single crystal X-ray diffraction. The molecular structure of **3Th** is shown in Figure 1, and further crystallographic details of **3Th** and **3U** can be found in Figures S1 and S2 and Table S1. In both cases, Sb_11_^3–^ cores are found with three coordinated {An(Tren^TIPS^)}^+^ fragments. That the Sb_11_^3–^ cores are found in a coordinated state is notable, because in all other examples of molecular Sb_11_^3–^ this Zintl trianion is found in its free form, as part of separated ion quadruples with noncoordinating cations such as [P(Me)(^*n*^Bu)_3_]^+^, [M(2.2.2-crypt)]^+^ (M = Na, K), [K(18C6)(NH_3_)_2_]^+^, and [Li(12C4)_2_]^+^ (12C4 = 12-crown-4 ether).^22−28^ Indeed, apart from lanthanide-stabilized Sb_8_^4–^,^18^ coordination of high nuclearity homonuclear Sb–Zintls to electropositive metals is generally rare or found in binary materials, e.g. M_3_Sb_11_ (M = group 1 metal),^34^ or intermetalloid clusters, e.g., [Ln@Sb_12_]^3–^,^35^ rather than discrete molecular complexes.^29−31^

For **3Th**, the Th–Sb distances span the range of 3.2978(11)–3.3427(1) Å (av. 3.3142 Å), which is slightly longer than the sum of the single bond covalent radii of Th and Sb (3.15 Å),^36^ and can be compared to Th–Sb bond lengths of 3.2849(3) Å in [Th(Tren^TIPS^){Sb(SiMe_3_)_2_}],^13^ 3.2282(2) Å in [{Th(Tren^TIPS^)}_2_(μ-SbH)],^17^ 3.0611(2) Å in [K(18C6)][{Th(Tren^TIPS^)}_2_(μ-Sb)],^17^ 3.0554(2) Å in [Th(Tren^TCHS^)(μ-SbH)K(18C6)],^17^ 3.0729(4) Å in [K(2.2.2-cryptand)][Th(Tren^TCHS^)(SbH)],^17^ and an average of 3.061 Å in [{Th(Tren^TIPS^)(μ-SbK_2_)}_4_].^17^ The Sb–Sb distances range from 2.7651(13) to 2.8424(15) Å (av. 2.8039 Å), in good agreement with double the sum of the single bond covalent radii of Sb (2.8 Å).^36^ The Th–N_amide_ and Th–N_amine_ bond lengths average 2.293 and 2.679 Å, respectively, and although they are toward the lower end of the range of triamidoamine Th–N_amide_ and Th–N_amine_ distances, they are unexceptional.^37−40^

For **3U**, the U–Sb bond lengths span the range of 3.2619(8)–3.3116(8) Å (average 3.2837 Å). This is slightly longer than the sum of the single bond covalent radii of U and Sb (3.1 Å),^36^ but compares to U–Sb distances of 3.2089(6) Å in [U(Tren^TIPS^){Sb(SiMe_3_)_2_}],^13^ 3.2437(8) Å in [U(Tren^DMBS^){Sb(SiMe_3_)_2_}] (Tren^DMBS^ = {N(CH_2_CH_2_NSiMe_2_^t^Bu)_3_}^3–^),^13^ and 3.3651(8) Å in [U(Tren^DMBS^){Sb(H)CH(SiMe_3_)_2_}].^16^ We note that the U–Sb distances in **3U** are ∼0.03 Å shorter than the Th–Sb bond lengths in **3Th**, but they would be predicted to be ∼0.05 Å shorter,^36^ which may simply be that the U–Sb distances cannot be any shorter due to the sterically demanding nature of Tren^TIPS^. The Sb–Sb bond lengths in **3U** range from 2.7673(1) to 2.8441(11) Å (av. 2.8025 Å), and show little difference to the corresponding metrics in **3Th**. The U–N_amide_ and U–N_amine_ bond lengths average 2.241 and 2.640 Å, respectively, and these metrics are typical of Tren-uranium(IV) distances.^41−47^

In prior examples of free Sb_11_^3–^ trianions,^22−28^ there are essentially two distinct groups of Sb–Sb distances. The two-coordinate Sb centers that each formally carry a negative charge tend to have Sb–Sb distances of ∼2.76 Å; the remaining three-coordinate Sb centers have Sb–Sb bond lengths of ∼2.83 Å, and the average Sb–Sb distances are ∼2.80 Å. It is interesting to note that the Sb–Sb distances in **3Th** and **3U** exhibit very similar average Sb–Sb distances and the pattern of short (∼2.76 Å) and longer (∼2.83 Å) Sb–Sb bonds is also found. This implies little impact on the Sb_11_^3–^ structures when free or coordinated, perhaps reflecting the largely electrostatic nature of the Th–Sb and U–Sb bonds in **3Th** and **3U**, respectively (see below).

### Spectroscopic Characterization

The ^1^H and ^13^C{^1^H} NMR spectra of **3Th** (Figures S3 and S4) are consistent with the diamagnetic formulation of **3Th**, though due to poor solubility we could not locate the ^29^Si{^1^H} resonance for this complex. The IR spectrum (Figure S9) is as expected, though it is not particularly informative since any Th–Sb and Sb–Sb absorptions would be expected to occur below the measurement cutoff. The UV/vis/NIR spectrum of **3Th** (Figure S11) exhibits two maxima centered at ∼15000 and ∼22500 cm^–1^, though the spectrum is clearly a composite of multiple overlapping and broad absorptions that are all effectively shoulders on a larger charge transfer feature. We modeled the UV/vis/NIR spectrum of **3Th** using time-dependent density functional theory (TDDFT, Figure S13) and a truncated model (**3Th′**, see below), which revealed a large number of absorptions over the range 12500–25000 cm^–1^ that are consistent with the experimental data. These absorptions correspond to charge transfer from occupied Th–Sb σ- and π-bonds, which are mainly of Sb character, see later, to vacant thorium 5f/6d hybrids in the virtual manifold. Similar low energy charge transfer features have been observed for related Th–Sb complexes,^17^ and these features are generally at lower energy than previously observed An–E (An = Th, U; E = P, As), similar to An–Sb, but higher energy than An–Bi absorptions with triamidoamine ancillary ligands.^13,16,17,20,21,33,38,42^

The ^1^H NMR spectrum of **3U** (Figure S6) spans the range from −43 to 61 ppm, which, qualitatively, is a relatively large range for uranium(IV), though this likely reflects the range of inequivalent H-atom environments in **3U**. However, the ^29^Si{^1^H} NMR spectrum of **3U** (Figure S7) revealed a resonance at 36.9 ppm, which is characteristic of uranium(IV).^48^ Again, the IR data (Figure S10) for **3U** were as expected, but not particularly informative. The UV/vis/NIR spectrum of **3U** (Figure S12) is similar to that of **3Th**, except that individual features are barely discernible because of a dominant charge transfer band across the spectrum.

### Computational Characterization

In order to provide greater insight into the electronic structures and bonding of **3Th** and **3U**, we performed DFT calculations (Tables S2 and S3). The size of these complexes, coupled to the presence of 14 heavy elements with *Z* ≥ 51 rendered geometry optimizations impracticable to perform, and to avoid program memory-limit issues the Si^i^Pr_3_ components were truncated to SiH_3_ units to produce molecules referred to as **3Th′** and **3U′**. Single point energy calculations were then performed, enabling Natural Localized Molecular Orbital (NLMO) and Quantum Theory of Atoms in Molecules (QTAIM) calculations to be performed, as shown in Table 1. Calculations used the BP86 TZP/ZORA (all-electron) level, since prior work has shown that this produces NLMO and QTAIM metrics that compare well to experimentally benchmarked hybrid-B3LYP calculations.^49−51^

**Table 1:** DFT, NLMO, and QTAIM Computed Properties of the U–Sb Bonds in **3Th′** and **3U′**^a^

	Bi^b^	charges^c^		NLMO^d^					QTAIM^e^			
cmpd	NM	Th	Sb	type	Th	Sb	7s/7p/6d/5f	5s/5p	ρ	∇^2^ρ	*H*	ε
**3Th**	0.72	1.90	–0.55	σ	6	91	14/0/68/18	77/20	0.03	0.03	–0.01	0.13
				σ	5	92	10/0/71/19	79/21	0.03	0.02	–0.01	0.13
				σ	6	91	12/0/70/18	78/22	0.03	0.03	–0.01	0.16
				π	15	79	28/0/53/19	7/93				
				π	15	79	32/0/50/18	7/93				
				π	15	79	26/0/55/19	6/94				
**3U**	1.07	1.84	–0.54	σ	6	92	15/0/63/22	78/22	0.03	0.03	–0.01	0.08
				σ	5	92	13/0/63/24	78/22	0.03	0.03	–0.01	0.14
				σ	6	92	15/0/63/22	78/22	0.03	0.03	–0.01	0.03
				π	14	79	24/0/47/29	8/92				
				π	14	79	24/0/47/29	8/92				
				π	13	79	25/0/47/28	7/93				

a Both compounds computed with density functional theory (DFT) at the BP86 TZP/ZORA (all-electron) level.

b Average Nalewajski–Mrozek bond indices.

c Average quadrupolar Multipole Derived Charge (MDC_q_).

d Natural Localized Molecular Orbital (NLMO) analysis.

e Quantum Theory of Atoms in Molecules (QTAIM) 3,–1 bond critical point topological electron density (ρ), Laplacian (∇^2^ρ), energy (*H*), and ellipticity (ε) analysis.

The computed An–Sb Nalewajski–Mrozek bond indices for **3Th′** and **3U′**, Table 1, are 0.72 and 1.07, respectively, which are consistent with polar-covalent single bonds. The MDC_q_ An/Sb charges for **3Th′** and **3U′** (Table 1) are 1.90/–0.55 and 1.84/–0.54, respectively. When taken together, these data suggest slightly more covalent U–Sb than Th–Sb linkages, though the differences are modest. The MDC_m_ spin densities in **3U′** average 2.41 and −0.16 for the U and Sb donor centers, respectively. These values are consistent with the presence of 5f^2^ uranium(IV) ions that have a net receipt of electron density from the ligands and the Sb ions being net electron donors.

**Figure 2:**
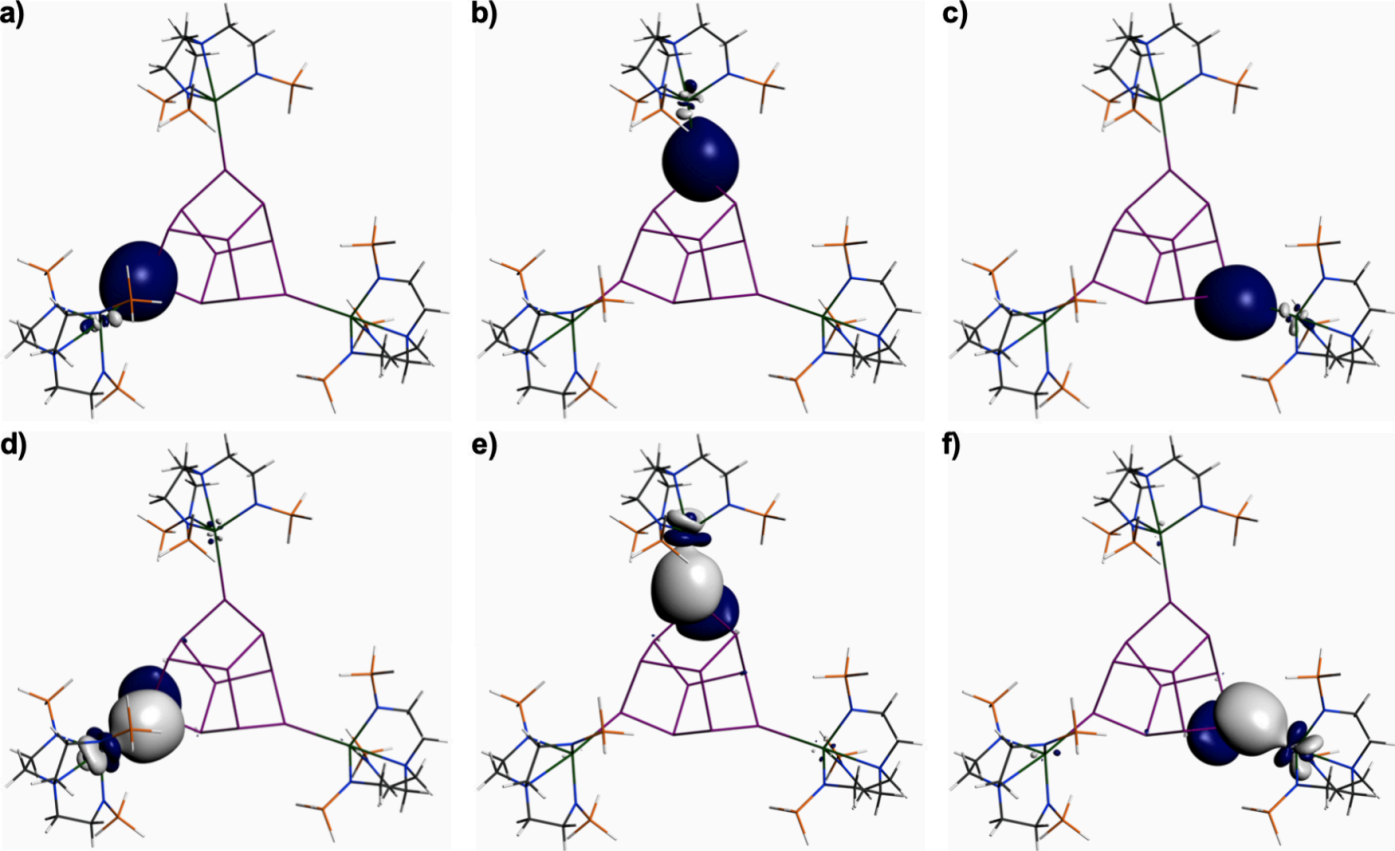
Natural Localized Molecular Orbitals for truncated **3U** (**3U′**). (a)–(c): U–Sb σ-bonds. (d)–(f): U–Sb π-bonds.

The NLMO data (Table 1) reveal very polarized An–Sb bonds, and the NLMOs for **3U′** are shown in Figure 2 (and see Figures S14 and S15 for details of **3Th′** and **3U′**). The NLMO analysis reveals σ-bonds dominated by Sb character (91–92%), with An contributions of only 5–6%. Interestingly, for both **3Th′** and **3U′** the An ion components exhibit significant 7s (10–15%) and 5f (18–25%) contributions, but the bonding is overwhelmingly dominated by 6d character (63–68%). The Sb lone pairs that engage in σ-donation are predominantly of 5s character (77–79%), with directionality afforded by 5p contributions (20–22%). In contrast, the An–Sb π-bonds exhibit larger An contributions (13–15%), but Sb contributions (79%) still dominate. Interestingly, for both **3Th′** and **3U′** the An ions exhibit approximately doubled (24–32%) 7s contributions compared to the An–Sb σ-bonds. The 5f character (18–29%) remains little changed compared to the An–Sb σ-bonds, with the balance still being the dominant 6d character (47–55%). It is notable that overall the uranium ions in **3U′** exhibit more 5f character and less 6d character than the thorium ions in **3Th′**, but the variance is remarkably low. Certainly, while the bonding is what would be expected for thorium, the 5f contributions for uranium are unexpectedly low considering its bonding is usually dominated by 5f over 6d character.^7^

Last, QTAIM analysis (Table 1) of **3Th′** and **3U′** provides a topological view of the bonding that complements the orbital-based NLMO method. In all cases, An–Sb 3,–1 bond critical points were located, and these were found to be polar, indeed rather ionic, in nature with low ρ and *H* terms. The ε values for the An–Sb bonds reflect that the An–Sb bonds are “slipped”, that is the antimony σ-bonding orbitals are not perfectly aligned to the An centers, and also the dative π-donations.^52,53^

## Conclusions

To conclude, although cesium antimonide **1** does not undergo protonolysis reactions with triamidoamine cyclometallate thorium and uranium complexes, it does engage in salt elimination with triamidoamine thorium and uranium separated ion pair complexes to afford the triactinide undeca-antimontriide Zintl clusters **3Th** and **3U**. Clusters **3Th** and **3U** clearly form via dehydrocoupling, providing two new, structurally authenticated, examples of the Sb_11_^3–^ Zintl trianion with only seven structurally authenticated examples reported previously. Complexes **3Th** and **3U** constitute unprecedented examples of molecular Sb_11_^3–^ being coordinated to anything, since all previous reports feature isolated Sb_11_^3–^ Zintl trinions in separated ion quadruple formulations. Quantum chemical calculations suggest largely ionic An–Sb interactions in **3Th** and **3U**, though the data suggest that the latter exhibits slightly more covalent An–Sb linkages than the former. Complexes **3Th** and **3U** bring a new formulation of the Sb_11_^3–^ Zintl trianion to the field of Zintl chemistry. We are currently further exploring the chemistry of the H_2_Sb^1–^ anion with the f-elements and will report on this in due course.

## Experimental Section

***Caution:** Depleted uranium (primary isotope ^238^U) is a weak α-emitter (∼4.4 MeV, *t*_1/2_ = 4.47 × 10^9^ years). Manipulations and reactions should be carried out under inert conditions in a designated uranium laboratory equipped with appropriate detection equipment (α- and β-counters) and a monitoring regime. Although the reactions that generate **3Th** and **3U** produce relatively large number of equivalents of H_2_, on submmole scales this can be safely vented to inert atmosphere exhaust systems.*

### General Considerations

All manipulations were carried out using Schlenk techniques or an MBraun UniLab glovebox under dry N_2_. Solvents were dried by passage through activated alumina towers and degassed before use. All solvents were stored over K- mirrors except for ethers, which were stored over activated 4 Å sieves. Deuterated solvent was distilled from K, degassed by three freeze–pump–thaw cycles, and stored under N_2_. The compounds [Cs(18C6)_2_][SbH_2_] (**1**)^19^ and [An(Tren^TIPS^)(L)][BPh_4_] (An = Th, L = DME (**2Th**); An = U, L = THF (**2U**))^20,21^ were prepared using literature methods.

### Instrumentation

Single crystals were examined on a Rigaku XtalLAB Synergy-S diffractometer equipped with a HyPix 6000HE photon counting pixel array detector with mirror-monochromated Cu Kα (λ = 1.54184 Å) or Mo Kα (λ = 0.71073 Å) radiation. Intensities were integrated from a sphere of data recorded on narrow (0.5°) frames by ω rotation. Cell parameters were refined from the observed positions of all of the strong reflections in each data set. Gaussian grid face-indexed absorption corrections with a beam profile correction were applied. The structures were solved by dual methods using SHELXT,^54^ and all non-hydrogen atoms were refined by full-matrix least-squares on all unique *F*^2^ values with anisotropic displacement parameters, with exceptions noted in the respective crystallographic information files. Except where noted, H atoms were refined with constrained geometries and riding thermal parameters; *U*_iso_(H) was set at 1.2 (1.5 for methyl groups) times the *U*_eq_ of the parent atom. The largest features in final difference syntheses were close to heavy atoms and were of no chemical significance. CrysAlisPro was used for control and integration,^55^ and SHELXL and Olex2 were employed for structure refinement.^56,57^ ORTEP-3 and POV-Ray were employed for molecular graphics.^58,59^^1^H, ^13^C{^1^H}, and ^29^Si{^1^H} NMR spectra were recorded on a Bruker 400 spectrometer operating at 400, 101, and 79 MHz, respectively; chemical shifts are quoted in ppm and are relative to TMS (^1^H, ^13^C, and ^29^Si). ATR-IR spectra were recorded on a Bruker Alpha spectrometer with a Platinum-ATR module in the glovebox. UV/vis/NIR spectra were recorded on a PerkinElmer LAMBDA 750 spectrometer. Data were collected in a 1 mm path-length cuvette loaded in a glovebox and were run versus the appropriate solvent.

### Synthesis and Isolation of [{Th(Tren^TIPS^)}_3_(μ_3_-Sb_11_)] (**3Th**)

Dark red **1** (0.32 g, 0.40 mmol) was added portion-wise to a colorless stirring slurry of **2Th** (0.50 g, 0.40 mmol) in benzene (20 mL). During the addition, the mixture turned into a red-brown suspension, accompanied by substantial gas evolution, and then the mixture was stirred for 30 min with the exclusion of light. The mixture was filtered to give a red-brown solution, and volatiles were removed *in vacuo* to yield a dark brown oily solid which was washed with Et_2_O (2 × 2 mL) to afford **3Th** as dark brown/black solid. Black crystals of **3Th** suitable for single-crystal X-ray diffraction studies were grown by the slow diffusion of pentane into a benzene solution at room temperature. Yield: 0.05 g, 34% (by Sb content). Anal. Calcd for C_99_H_225_N_12_Sb_11_Si_9_Th_3_: C, 30.71; H, 5.86; N, 4.34%. Found: C, 30.96; H, 5.90; N, 4.23%. ^1^H NMR (D_8_-THF, 298 K): δ 1.05–1.41 (m, 189H, C*H*(CH_3_)_2_ and CH(C*H*_3_)_2_), 2.92–2.99 (br, 18H, NCH_2_C*H*_2_), 3.68–3.88 (br, 18H, NC*H*_2_CH_2_) ppm. ^13^C{^1^H} NMR (D_8_-THF, 298 K): δ 71.51 (*C*H_2_), 54.73 (*C*H_2_), 21.79 (CH(*C*H_3_)_2_), 17.17 (*C*H(CH_3_)_2_) ppm. ^29^Si{^1^H} NMR (D_8_-THF, 298 K): not observed due to the poor solubility of the compound once isolated. ATR-IR ν/cm^–1^: 2937 (m), 2886 (m), 2859 (s), 1457 (m), 1380 (w), 1362 (w), 1271 (w), 1244 (w), 1136 (w), 1111 (w), 1041 (m), 1009 (w), 925 (s), 879 (s), 808 (s), 721 (vs), 621 (s), 630 (m), 551 (w), 516 (w), 443 (w).

### Synthesis and Isolation of [{U(Tren^TIPS^)}_3_(μ_3_-Sb_11_)] (**3U**)

Dark red **1** (0.32 g, 0.40 mmol) was added portion-wise to a green-yellow stirring slurry of **2U** (0.50 g, 0.4 mmol) in benzene (20 mL). During the addition, the mixture turned into a dark red-brown suspension, accompanied by gas evolution, and then the mixture was stirred for 24 h with the exclusion of light. The mixture was then hot filtered (100 °C) through Celite to give a dark red-brown solution. Volatiles were removed *in vacuo* to yield a dark brown oily solid (^1^H NMR of this crude product revealed the presence of a significant amount of [U(Tren^TIPS^)]), which was washed with Et_2_O (2 × 2 mL) to remove the [U(Tren^TIPS^)], and dried *in vacuo,* affording **3U** as a small crop of dark brown/black solid. Red crystals of **3U** suitable for single-crystal X-ray diffraction studies were grown by the slow evaporation of a THF solution at room temperature. Yield: <5% (by Sb content). Starting the reaction cold (−78 °C) does not affect the outcome of the reaction. Anal. Calcd for C_99_H_225_N_12_Sb_11_Si_9_U_3_: C, 30.57; H, 5.83; N, 4.32%; Found: C, 30.40; H, 5.78; N, 4.21%. ^1^H NMR (D_8_-THF, 298 K): δ 60.99 (br), 15.58 (br), 13.94 (br), 12.68 (br), 10.72 (br), 7.32 (br), 5.24 (br), −0.60 (br), −5.50 (br), −12.86 (br), −13.27 (br), −28.88 (br), −40.46 (br), −42.71 (br) ppm. ^29^Si{^1^H} NMR (D_8_-THF, 298 K): δ 36.92 ppm. ATR-IR ν/cm^–1^: 2915 (m), 2859 (m), 1459 (m), 1426 (w), 1379 (w), 1351 (w), 1105 (m), 1057 (s), 925 (s), 880 (m), 730 (vs), 704 (s), 671 (m), 632 (w), 544 (w), 513 (w), 442 (w). Due to the low yield of this reaction, this compound was not characterized by SQUID magnetometry.

### Computational Details

Due to the size of **3Th** and **3U**, full geometry optimizations were not practicable to perform, and to avoid NBO memory-limit issues the Si^i^Pr_3_ components were truncated to SiH_3_ units to produce **3Th′** and **3U′**. Therefore, we used coordinates derived from their crystal structures as the starting points and geometry optimized the H atom positions while freezing the relative positions of all other atoms. No other constraints were imposed on the structures during the geometry optimizations. The calculations were performed using the Amsterdam Density Functional (ADF) suite version 2017 with standard convergence criteria.^60,61^ The DFT calculations employed Slater type orbital (STO) triple-ζ-plus polarization all-electron basis sets (from the Dirac and ZORA/TZP databases of the ADF suite). Scalar relativistic approaches (spin–orbit neglected) were used within the ZORA Hamiltonian^62−64^ for the inclusion of relativistic effects and the local density approximation (LDA) with the correlation potential due to Vosko et al. was used in all of the calculations.^65^ Generalized gradient approximation (GGA) corrections were performed using the functionals of Becke and Perdew.^66,67^ Natural Bond Order (NBO) and Natural Localized Molecular Orbital (NLMO) analyses were carried out with NBO 6.0.19.^68^ The Quantum Theory of Atoms in Molecules analysis^69,70^ was carried out within the ADF program. We quote Nalewajski–Mrozek bond orders since they reproduce expected bond multiplicities reliably in polar heavy atom structures whereas Mayer bond orders for polar bonds often do not conform with chemical intuition.^71^ The ADF-GUI (ADFview) was used to prepare three-dimensional plots of the electron density. The TDDFT spectrum of **3Th′** was modeled using the SAOP functional using a THF solvent continuum as implemented in the ADF program.

## Supporting Information Available

Experimental and computational procedures; crystallographic details for **3Th** and **3U**; NMR, IR, and UV/vis/NIR spectroscopic details for **3Th** and **3U**; computational data for **3Th′** and **3U′** (PDF)
