# Supplementary material for: f-Element Zintl Chemistry: Actinide-Mediated Dehydrocoupling of H2Sb1– Affords the Trithorium and Triuranium Undeca-Antimontriide Zintl Clusters [{An(TrenTIPS)}3(μ3-Sb11)] (An = Th, U; TrenTIPS = {N(CH2CH2NSiiPr3)3}3–)
Source: Inorg Chem. 2024 May 20;63(43):20153–60. doi: 10.1021/acs.inorgchem.4c00923 (PMC11523227; doi:10.1021/acs.inorgchem.4c00923)
Supplement: Supplementary file 1 — ic4c00923_si_001.pdf [file ic4c00923_si_001.pdf]

– Supporting Information –

f-Element Zintl Chemistry: Actinide-Mediated Dehydrocoupling of H_2Sb^{1-} Affords the Tri-Thorium and -Uranium Undeca-Antimontriide Zintl Clusters [{An(Tren^{TIPS})}$_3$(μ_3-Sb_{11})] (An = Th, U; Tren^{TIPS} = {N($CH_2CH_2NSi^iPr_3$)$_3$}$^{3-}$)

Jingzhen Du,[1,2] Kevin Dollberg,[3] John A. Seed,[1] Ashley J. Wooles,[1] Carsten von Hänisch,[3]* and Stephen T. Liddle[1]*

[1] Department of Chemistry and Centre for Radiochemistry Research, The University of Manchester, Oxford Road, Manchester, M13 9PL, UK.

[2] Present address: College of Chemistry, Zhengzhou University, Zhengzhou, 450001, China.

[3] Fachbereich Chemie, Philipps-Universität Marburg, Hans-Meerwein-Straße 4, 35043 Marburg, Germany.

*Email: haenisch@chemie.uni-marburg.de; steve.liddle@manchester.ac.uk

Figures

Molecular Structures

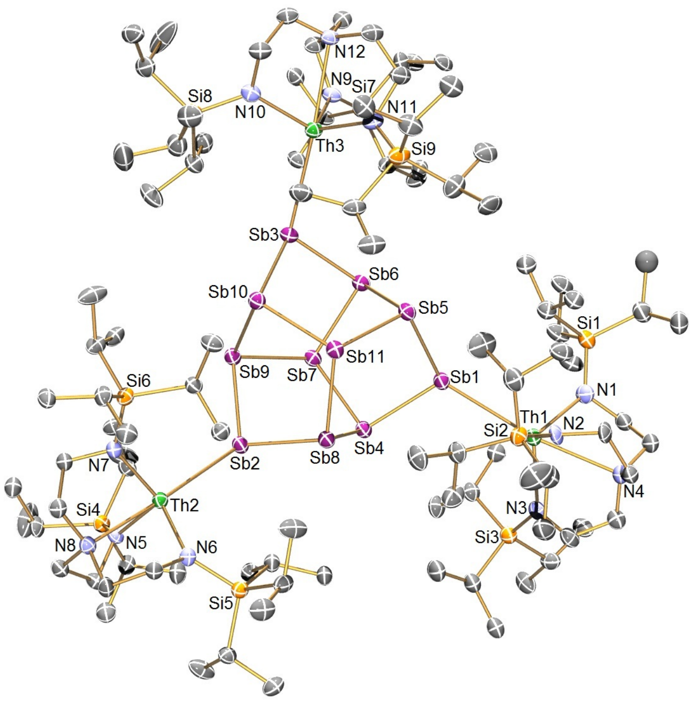

Figure S1. Molecular structure of **3Th** at 100 K with displacement ellipsoids set at 40%. Hydrogen atoms, disordered components, and lattice solvent molecules are omitted for clarity.

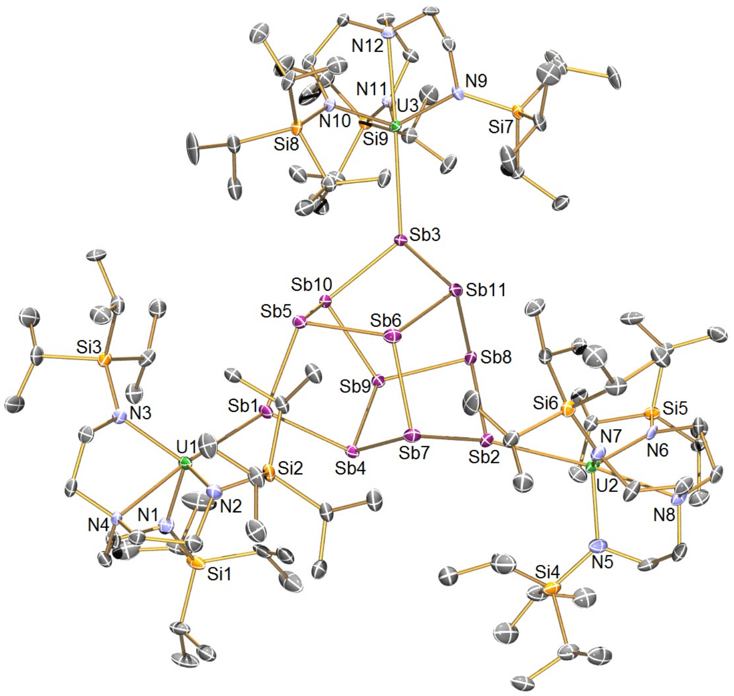

Figure S2. Molecular structure of **3U** at 130 K with displacement ellipsoids set at 40%. Hydrogen atoms, disordered components, and lattice solvent molecules are omitted for clarity.

NMR Spectra

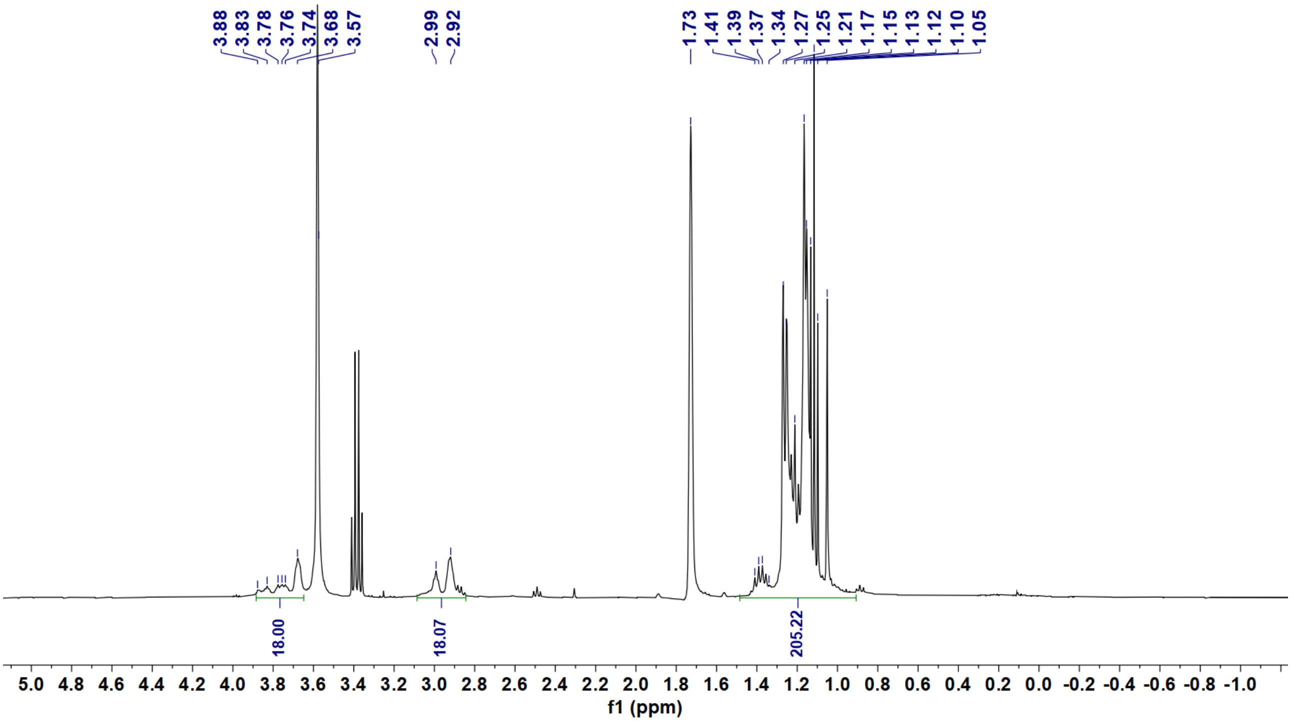

Figure S3. ^{1}H NMR (D_8-THF, 298 K) spectrum of **3Th**.

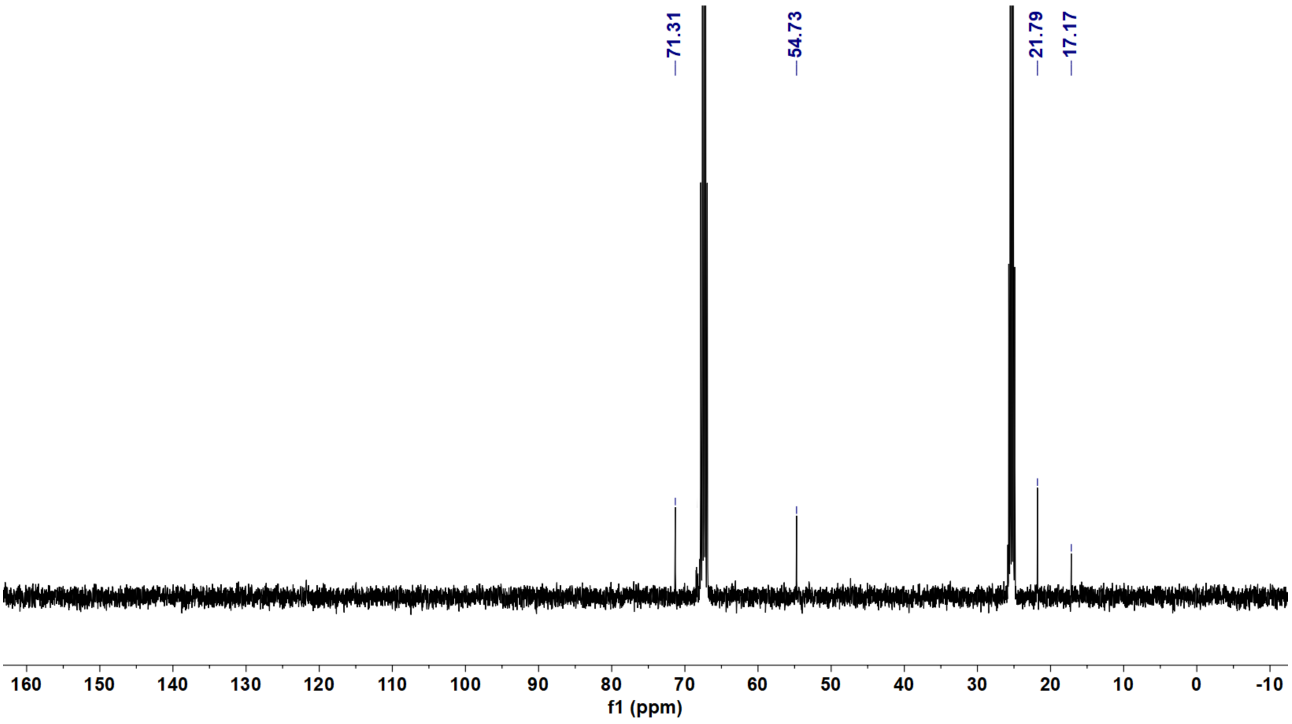

Figure S4. $^{13}C\{^{1}H\}$ NMR (D_8-THF, 298 K) spectrum of **3Th**.

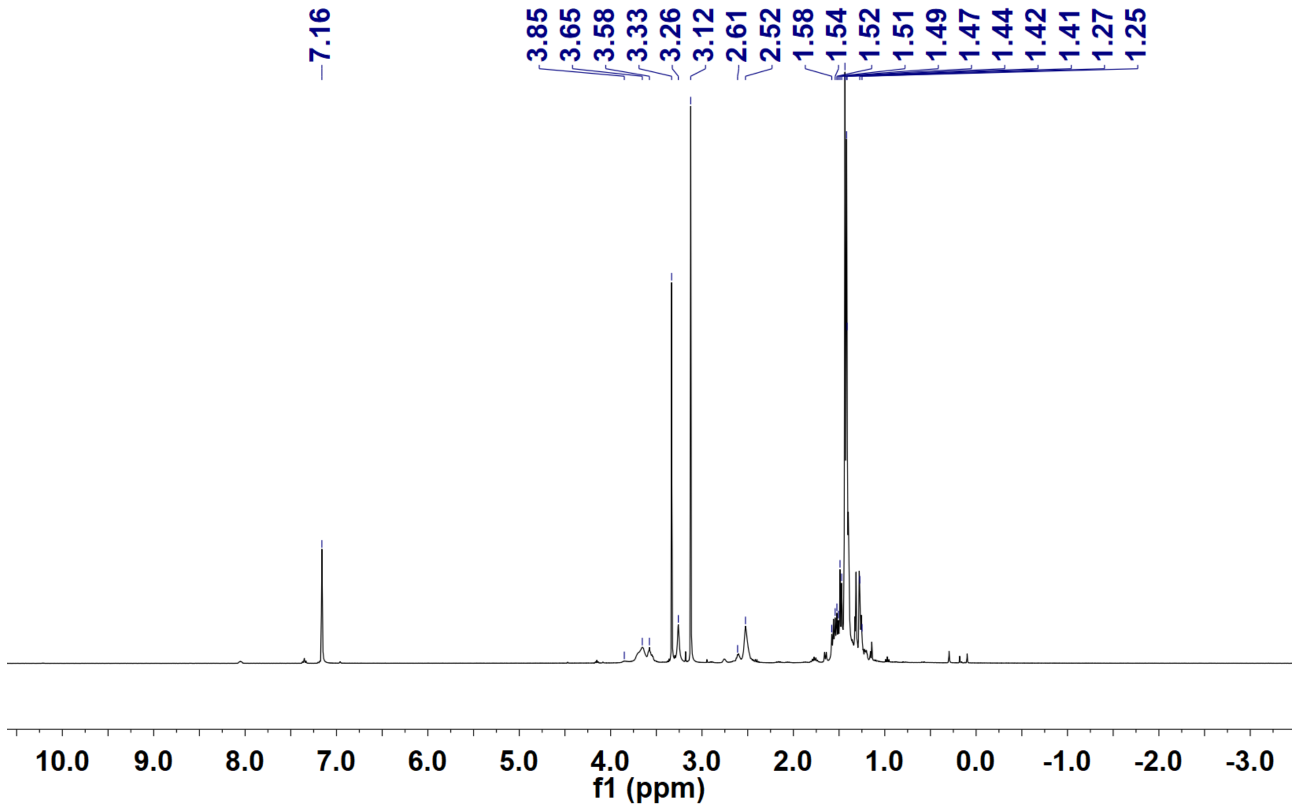

Figure S5. ^{1}H NMR (C_6D_6, 298 K) spectrum of the crude reaction mixture that produces **3Th**. Some resonances of **3Th** are shifted compared to the pure material recorded in D_8-THF.

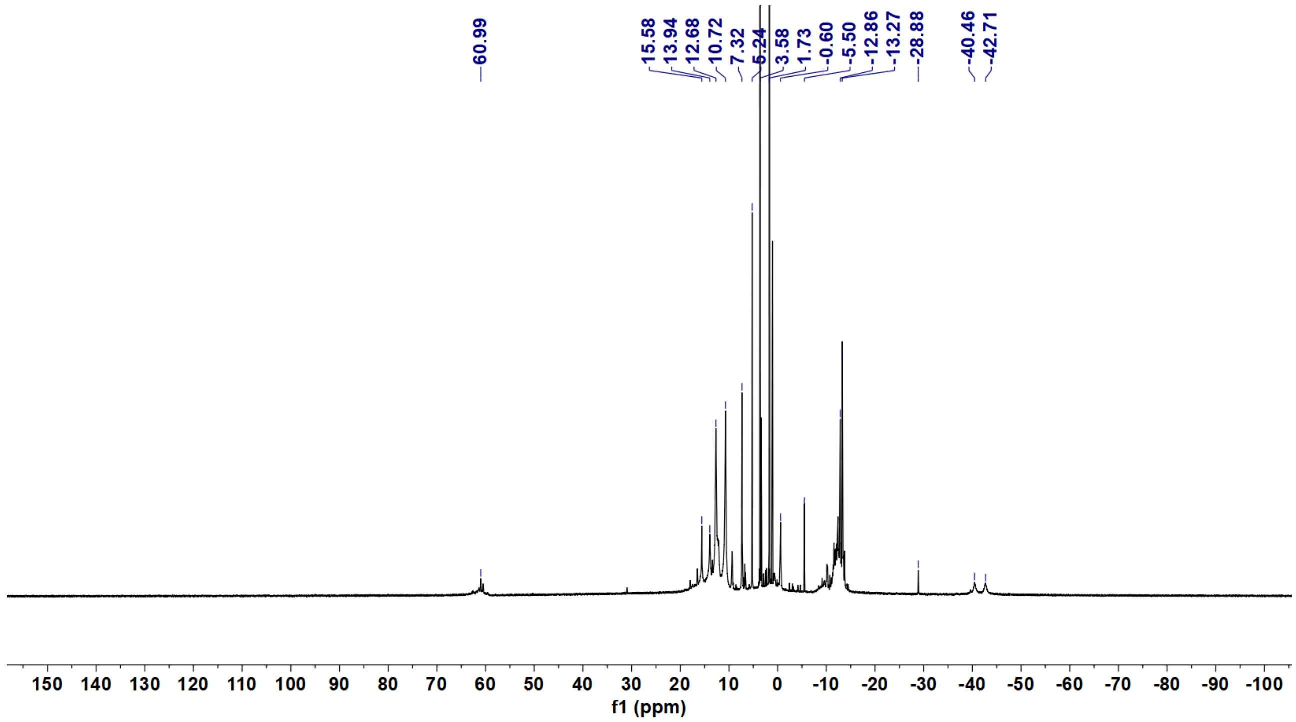

Figure S6. ^{1}H NMR (D_8-THF, 298 K) spectrum of **3U**.

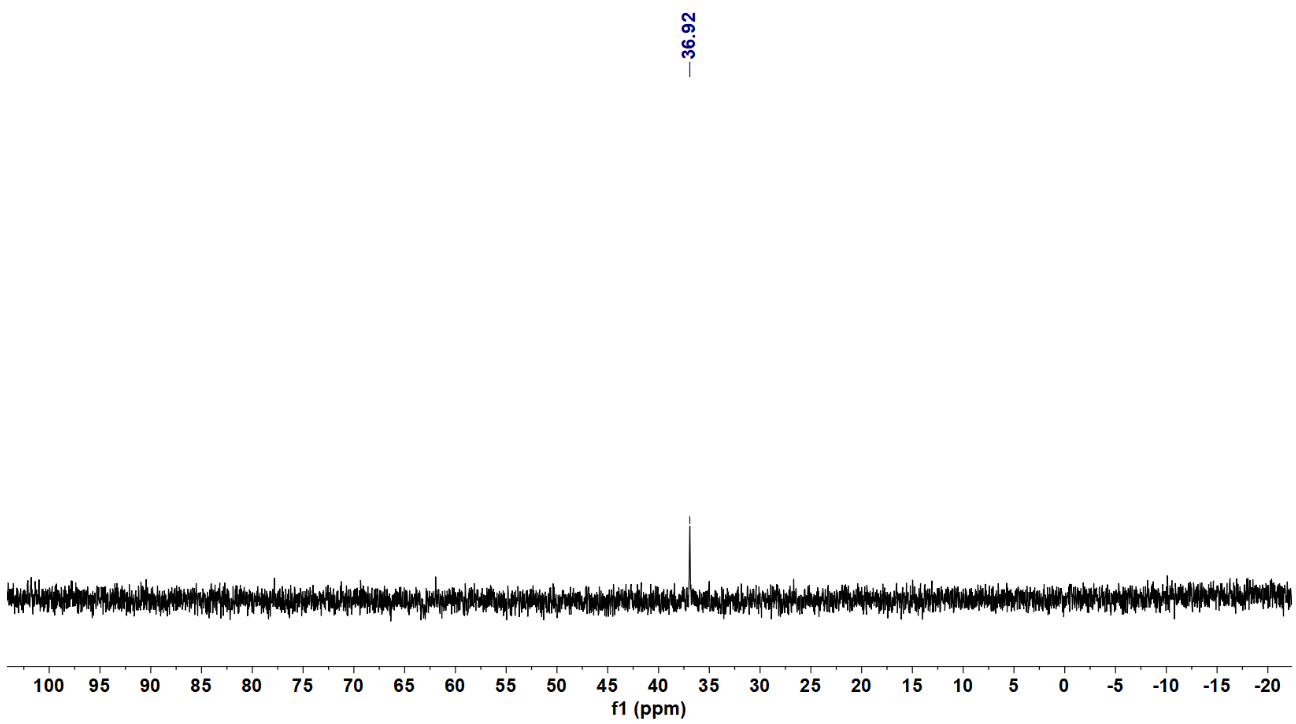

Figure S7. $^{29}Si\{^{1}H\}$ NMR (D_8-THF, 298 K) spectrum of **3U**.

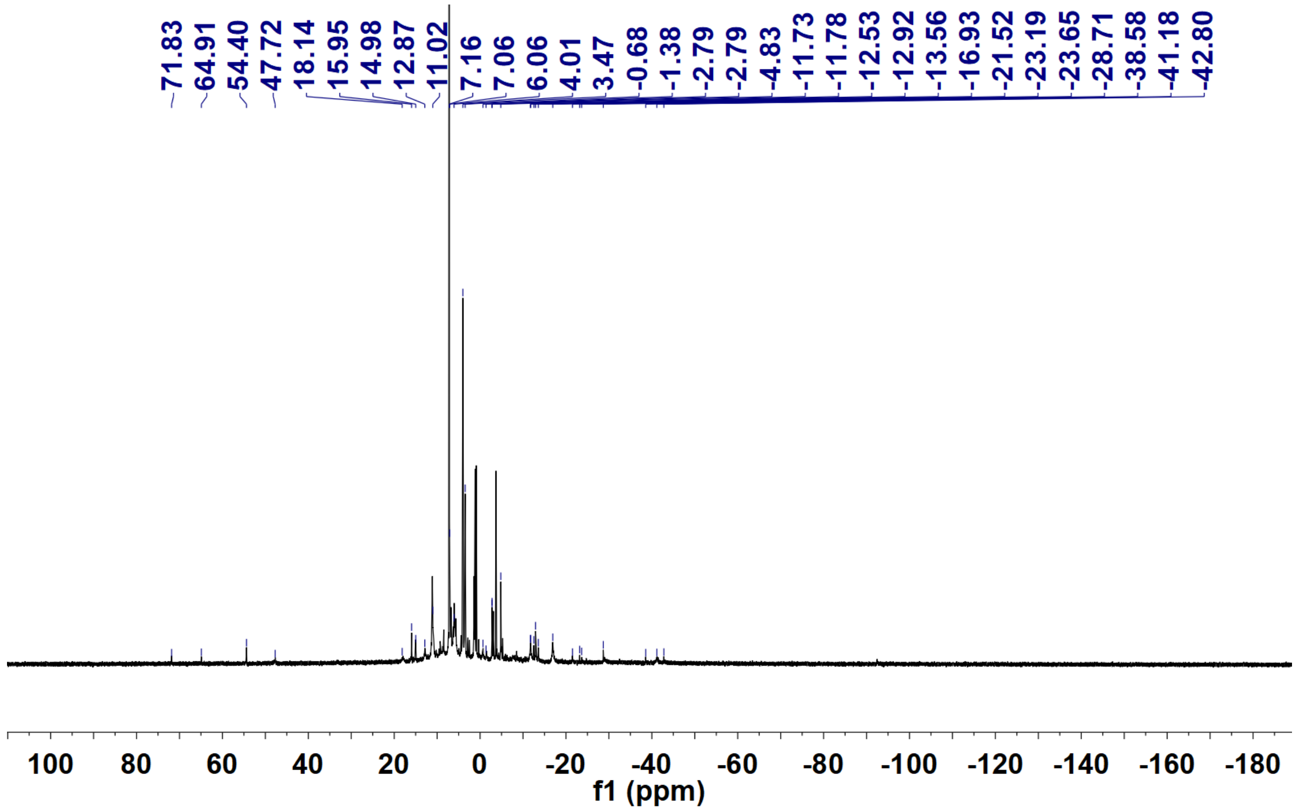

Figure S8. ^{1}H NMR (C_6D_6, 298 K) spectrum of the crude reaction mixture that produces **3U**. Some resonances of **3U** are shifted compared to the pure material recorded in D_8-THF.

ATR-IR Spectra

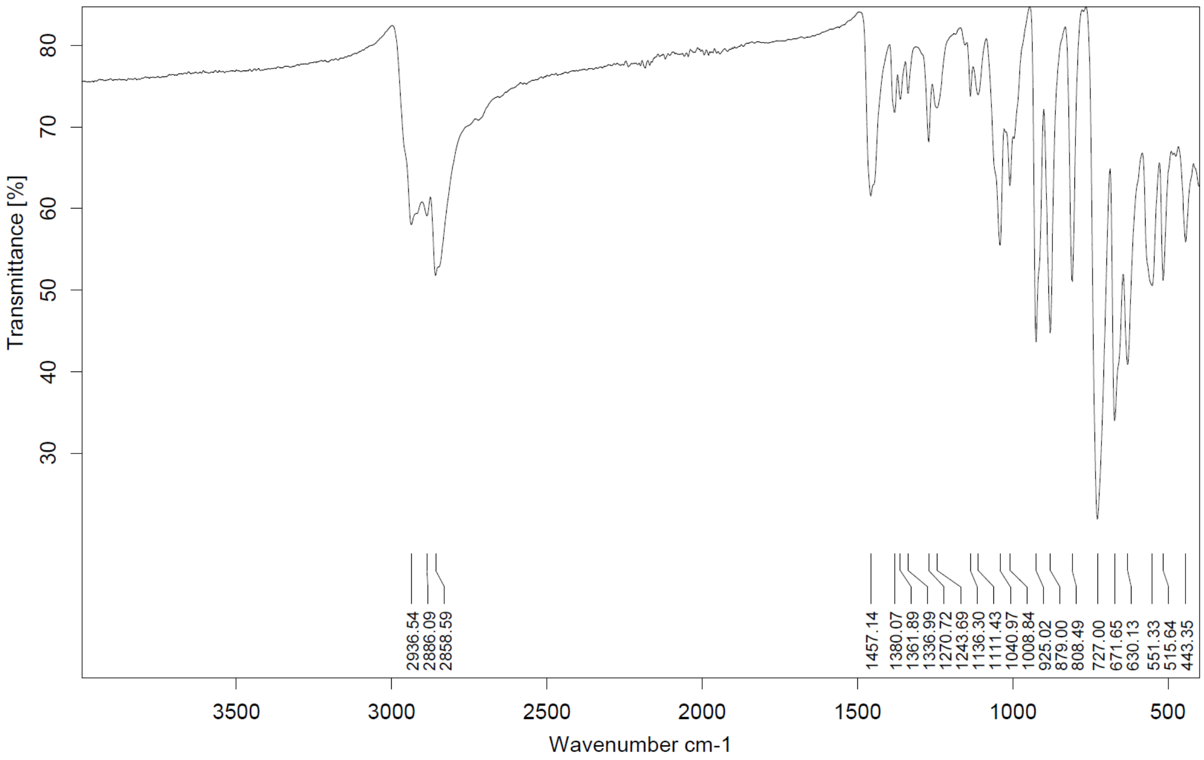

Figure S9. ATR-IR spectrum of **3Th**.

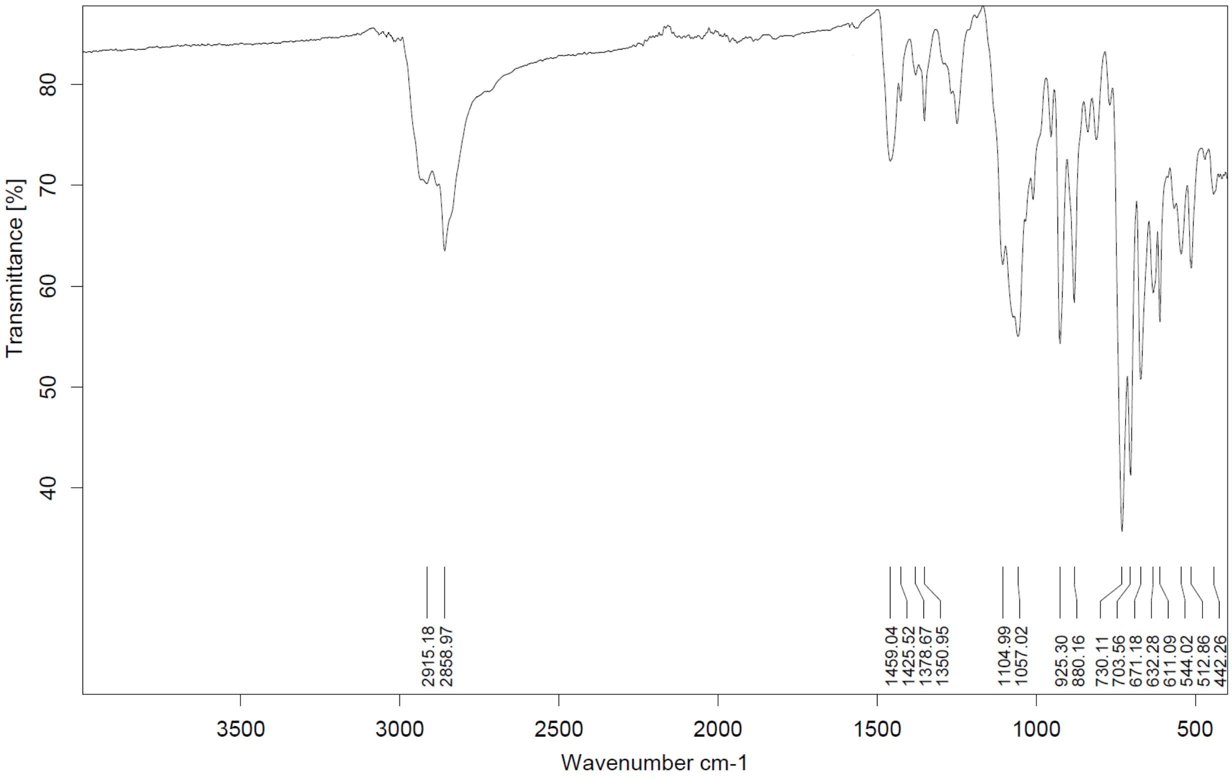

Figure S10. ATR-IR spectrum of **3U**.

UV/Vis/NIR Spectra

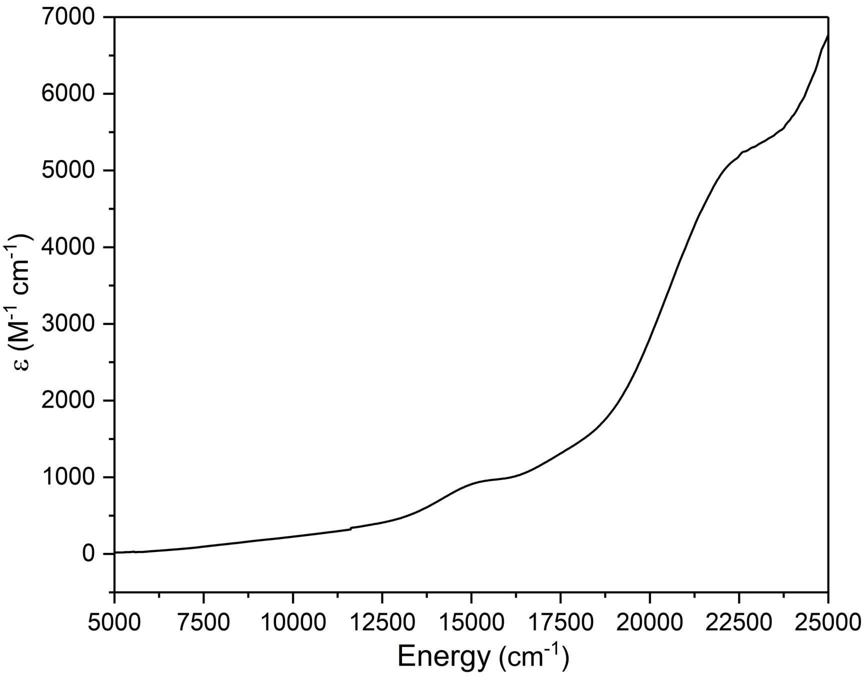

Figure S11. UV/Vis/NIR spectrum of **3Th** recorded in a saturated solution in THF (~5 mM), over the range 5000-25000 cm^{-1}.

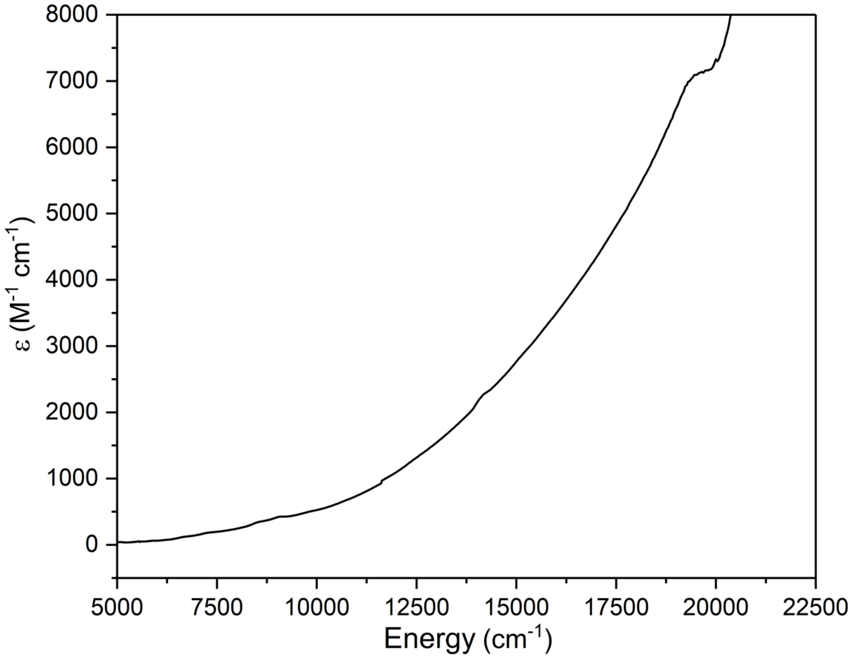

Figure S12. UV/Vis/NIR spectrum of **3U** recorded in a saturated solution in THF (~5 mM), over the range 5000-22500 cm^{-1}.

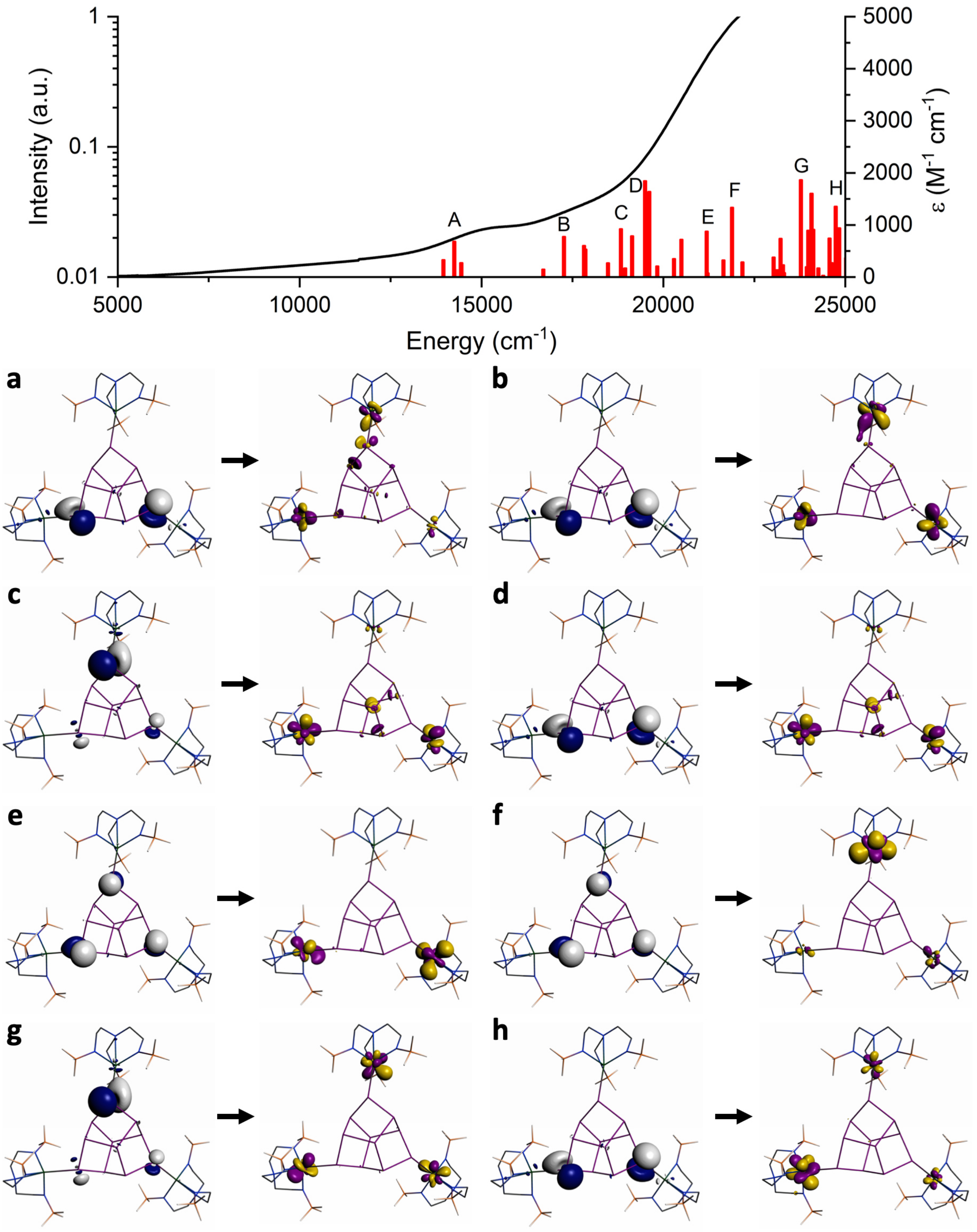

Figure S13. UV/Vis/NIR spectrum of **3Th'** recorded in a saturated solution in THF (~5 mM) over the range 5000-25000 cm^{-1} with simulated absorptions derived from TD-DFT represented as vertical oscillator strength red lines and below (a-h) representative electronic transitions.

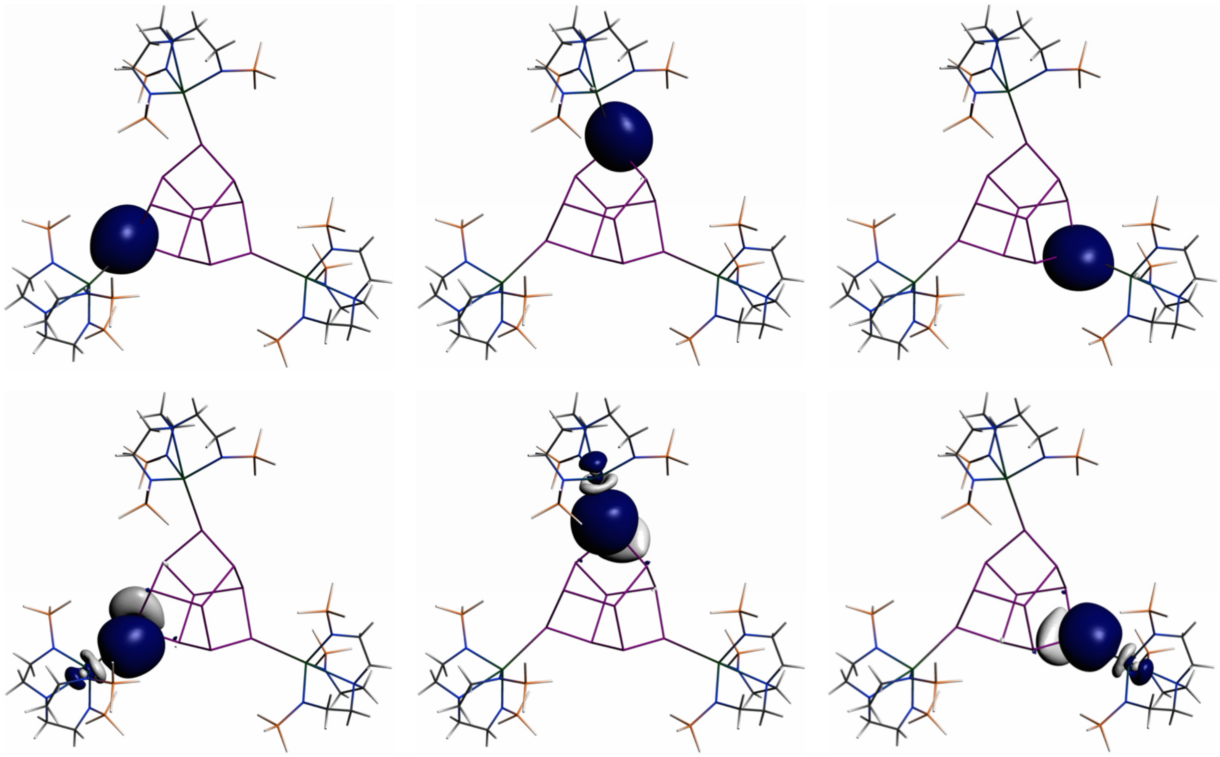

Figure S14. Natural Localized Molecular Orbitals for **3Th'**. Top: Th-Sb σ-bonds. Bottom: Th-Sb π-bonds.

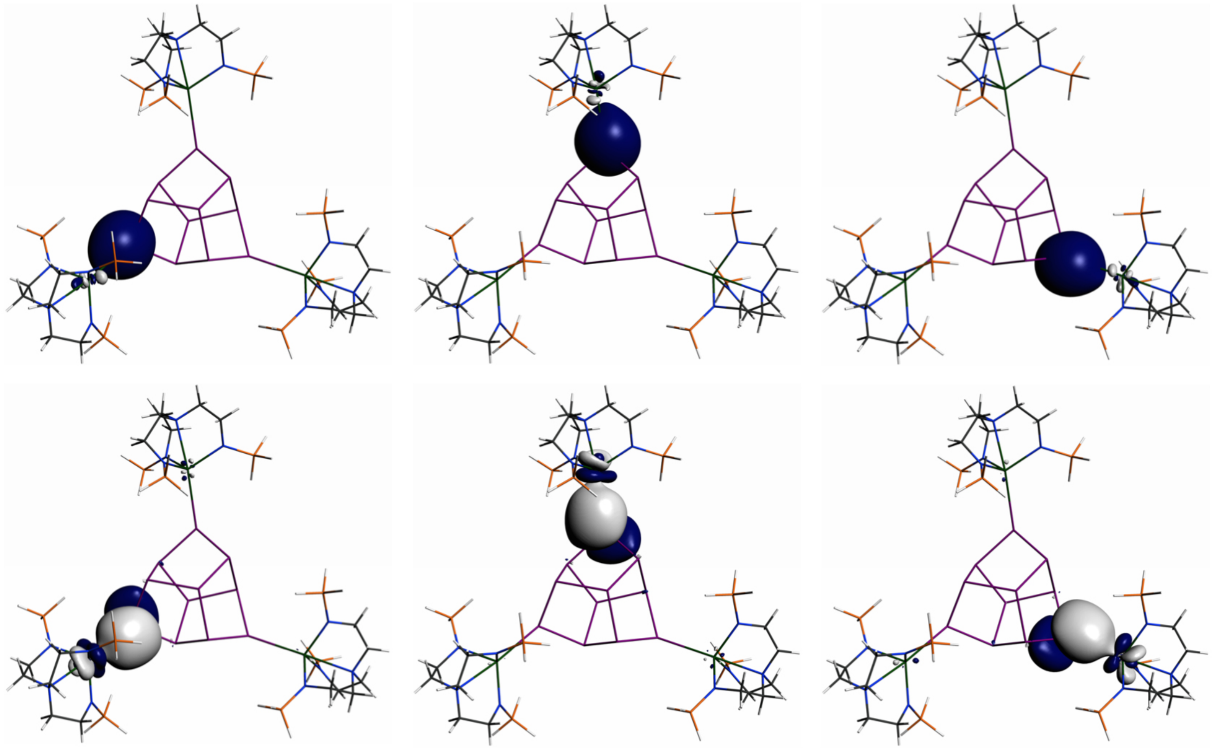

Figure S15. Natural Localized Molecular Orbitals for **3U'**. Top: U-Sb σ-bonds. Bottom: U-Sb π-bonds.

Tables

Table S1. Crystallographic data for 3Th and 3U

	**3Th**	**3U**
Formula	$C_{111}H_{237}N_{12}Sb_{11}Si_9Th_3$	$C_{119}H_{265}N_{12}O_5Sb_{11}Si_9U_3$
Fw, g mol^{-1}	4028.29	4250.56
Cryst size, mm	0.240 × 0.121 × 0.111	0.178 × 0.104 × 0.094
Crystal system	monoclinic	monoclinic
Space group	$P2_1$/c	$P2_1$/c
Collection Temperature (K)	100(2)	130(2)
a, (Å)	27.4564(3)	28.3252(2)
b, (Å)	21.3793(2)	20.8079(2)
c, (Å)	25.7408(2)	26.7795(2)
α, (°)	90	90
β, (°)	90.9530(10)	93.1450(10)
γ, (°)	90	90
V, (Å^3)	15107.7(2)	15759.7(2)
Z	4	4
ρ_{calc} g cm^{-3}	1.771	1.791
μ, mm^{-1}	25.694	5.037
No. of reflections measured	121319	223404
No. of unique reflections, R_{int}	26628, 0.1455	42003, 0.0594
No. of reflections with $F^2 > 2\sigma(F^2)$	18986	35088
Transmission coefficient range	0.351-1.000	0.315-0.738
R, $R_w{}^a$ ($F^2 > 2\sigma(F^2)$)	0.0678, 0.1625	0.0694, 0.1526
R, $R_w{}^a$ (all data)	0.1036, 0.1892	0.0847, 0.1563
S^a	0.975	1.321
Parameters, Restraints	1370, 2487	1523, 775
Max., min. difference map, e Å^{-3}	2.114, –2.329	2.031, –1.759

[a] Conventional $R = \Sigma||F_o| - |F_c||/\Sigma|F_o|$; $R_w = [\Sigma w(F_o{}^2 - F_c{}^2)^2/\Sigma w(F_o{}^2)^2]^{1/2}$; $S = [\Sigma w(F_o{}^2 - F_c{}^2)^2/\text{no. data} - \text{no. params})]^{1/2}$ for all data.

Table S2. Coordinates and single point energy for 3Th'

1.C	3.498458	7.189904	-3.118544
2.C	6.529306	6.270217	-2.755982
3.C	4.706698	7.956707	-2.694962
4.C	3.909134	-7.299185	-2.475972
5.C	6.791753	4.876381	-2.113697
6.C	3.643530	-8.669603	-1.818724
7.C	1.208652	-8.917556	-1.841302
8.C	-7.911576	0.537317	-1.384531
9.C	0.023430	-8.121929	-1.362727
10.C	6.292790	7.736260	-0.874251
11.C	-8.874782	1.377113	-0.624416
12.C	2.448083	-9.413493	0.228423
13.C	5.410043	7.820975	0.411174

14.C	-9.391194	0.107819	1.345733
15.C	3.027103	-8.557412	1.350022
16.C	-9.183586	2.533476	1.457575
17.C	-8.056993	3.548240	1.495519
18.C	-8.679085	-0.368187	2.577102
19.H	5.775937	3.045932	-4.524274
20.H	-0.493490	-6.400446	-4.059954
21.H	6.070696	6.087016	-3.737906
22.H	5.262822	8.395249	-3.543246
23.H	2.867546	7.853653	-3.742048
24.H	3.801807	6.379500	-3.810128
25.H	-5.246676	0.359978	-3.314165
26.H	-6.289826	2.520810	-3.149820
27.H	4.812368	-7.379504	-3.107990
28.H	7.471095	6.830680	-2.924931
29.H	3.087702	-7.067907	-3.178491
30.H	0.204609	6.835275	-2.819369
31.H	1.404226	-8.644093	-2.888615
32.H	6.129068	-4.907602	-2.472870
33.H	3.588006	-9.488794	-2.558587
34.H	7.570645	4.358666	-2.700259
35.H	4.070994	2.154497	-3.026721
36.H	6.439480	1.660584	-2.673864
37.H	-8.107232	0.673145	-2.463591
38.H	4.415364	8.764365	-2.012008
39.H	0.006337	-4.418432	-2.733495
40.H	1.016016	-10.008119	-1.816224
41.H	-2.058380	-5.668216	-2.388278
42.H	-0.872252	-8.436158	-1.928294
43.H	1.074990	8.725299	-1.609156
44.H	6.608947	8.737220	-1.234177
45.H	4.469748	-8.883556	-1.127592
46.H	-4.297461	2.035825	-1.818917
47.H	7.216933	5.004638	-1.102700
48.H	-8.068893	-0.539146	-1.192208
49.H	-9.925682	1.140815	-0.897949
50.H	6.638791	-6.720413	-0.978723
51.H	-8.702777	2.421292	-0.927964
52.H	7.203640	7.197019	-0.580801
53.H	-0.201053	-8.367643	-0.309625
54.H	3.036699	-10.328257	0.036818
55.H	0.674008	6.622497	-0.432572
56.H	4.445550	8.312938	0.196870
57.H	1.424889	-9.705513	0.498547
58.H	5.633722	-4.682904	-0.092136
59.H	-7.856173	3.921888	0.472794
60.H	-9.359381	-0.695197	0.595467
61.H	-10.083488	2.909213	0.937294
62.H	5.922166	8.451431	1.157292
63.H	4.053407	-8.241500	1.086674
64.H	-10.455483	0.345248	1.537963
65.H	-5.451275	-2.565656	1.889317

66.H	-8.429190	4.434704	2.045659
67.H	3.145047	-9.188918	2.249471
68.H	7.504721	6.408533	2.304866
69.H	-9.461980	2.286421	2.491330
70.H	-7.753223	-3.307758	2.267074
71.H	5.953975	4.539796	2.558581
72.H	-9.208498	-1.255017	2.969559
73.H	-5.789587	5.292699	2.984879
74.H	-8.738168	0.388967	3.379178
75.H	0.377813	-6.291526	3.183823
76.H	0.604234	-8.719133	3.331659
77.H	5.460394	6.723817	3.540444
78.H	-5.088859	3.194867	4.015308
79.H	-6.432994	-2.497707	4.116665
80.H	2.305856	-7.310037	4.284642
81.H	-7.298396	4.146193	4.475290
82.N	5.617074	7.005570	-1.926830
83.N	5.510257	4.132225	-2.083231
84.N	2.780540	6.661417	-1.968606
85.N	0.289705	-6.665487	-1.512341
86.N	4.033536	-6.252711	-1.440320
87.N	2.379998	-8.569220	-1.040563
88.N	-6.534838	0.946315	-1.000063
89.N	-8.689372	1.282156	0.801902
90.N	5.239783	6.417105	0.840965
91.N	2.148807	-7.391286	1.590036
92.N	-6.874712	2.960484	2.089770
93.N	-7.256137	-0.673983	2.243856
94.Sb	0.159281	0.113324	-1.104340
95.Sb	2.675548	-0.893834	-0.251388
96.Sb	-0.117196	2.602146	0.166583
97.Sb	-1.650950	-1.502293	0.332178
98.Sb	2.331954	3.218853	1.304157
99.Sb	2.232152	-3.092206	1.394953
100.Sb	3.160793	0.681469	2.025153
101.Sb	-3.031942	0.123272	2.125877
102.Sb	-0.147027	-2.276029	2.541475
103.Sb	-1.085764	2.000379	2.707572
104.Sb	0.862221	0.153147	3.588684
105.Si	5.454633	2.705965	-3.095677
106.Si	-0.596433	-5.787015	-2.690538
107.Si	-5.568578	1.467309	-2.358598
108.Si	1.138252	7.228669	-1.716641
109.Si	5.646075	-5.624874	-1.249445
110.Si	6.056196	6.014974	2.365080
111.Si	-6.703517	-2.306781	2.657360
112.Si	1.345693	-7.426001	3.141820
113.Si	-6.244730	3.937781	3.429292
114.Th	4.045653	5.215650	-0.690728
115.Th	2.093342	-6.008159	-0.231256
116.Th	-6.085434	1.001494	1.241867

Energy: −596.00676671 eV

Table S3. Coordinates and single point energy for 3U'

1.C	3.203815	-6.498931	-5.811739
2.C	4.663808	-6.562954	-5.558125
3.C	2.164351	8.132112	-3.864708
4.C	6.135132	-5.757054	-3.759500
5.C	5.905865	-4.254265	-3.745179
6.C	4.801877	-7.778826	-3.465279
7.C	3.033222	8.730229	-2.779912
8.C	4.295146	-7.648309	-2.057916
9.C	1.430167	9.400208	-1.051523
10.C	4.110490	6.872056	-0.592422
11.C	3.521235	8.276520	-0.448870
12.C	0.350761	8.738410	-0.249152
13.C	-7.549832	-1.003092	5.640014
14.C	-4.708307	-3.832521	5.994268
15.C	-7.133623	-1.925968	6.756284
16.C	-5.053985	-3.126592	7.285284
17.C	-4.041137	-0.134374	7.621948
18.C	-5.374127	-0.769263	7.953099
19.H	3.229844	-3.983598	-7.707997
20.H	0.976804	-4.780344	-7.417872
21.H	2.996999	-6.673132	-6.883319
22.H	5.153338	-5.715353	-6.058201
23.H	1.542962	5.714435	-6.135464
24.H	3.853771	6.097041	-5.574464
25.H	5.130260	-7.492235	-5.936662
26.H	1.738179	-2.828169	-6.159353
27.H	2.677689	-7.316177	-5.286758
28.H	2.600914	8.384498	-4.848151
29.H	6.932328	-6.064619	-4.465586
30.H	5.558539	-3.891587	-4.726416
31.H	4.090782	-8.393572	-4.034872
32.H	2.642355	4.250217	-4.530551
33.H	1.157616	8.583692	-3.853053
34.H	5.789218	-8.277266	-3.511612
35.H	6.859278	-3.735513	-3.543774
36.H	3.157710	9.824572	-2.891055
37.H	6.442393	-6.061894	-2.750647
38.H	4.026108	8.268377	-2.832676
39.H	-2.281234	8.998022	-2.066038
40.H	-2.306118	6.560453	-2.219635
41.H	0.971173	9.816650	-1.959873
42.H	4.211693	-8.662364	-1.624367
43.H	1.395597	-9.059575	-1.606127
44.H	5.026236	-7.114814	-1.426431
45.H	0.486959	-6.802964	-1.355683
46.H	5.904908	-1.734521	-1.578329
47.H	4.506121	6.700259	-1.604288
48.H	4.275868	9.074957	-0.585657
49.H	1.898994	10.243374	-0.505783
50.H	6.706437	-3.832760	-0.708989

51.H	-0.414433	9.489944	0.015736
52.H	4.967328	6.771535	0.096626
53.H	4.419889	-3.126917	-0.229147
54.H	-2.749617	7.689237	-0.101010
55.H	3.098166	8.369902	0.560552
56.H	0.746374	8.371461	0.715265
57.H	4.476977	3.830512	0.743685
58.H	1.977029	-7.743765	0.326568
59.H	-7.097400	-2.336125	1.958822
60.H	2.109282	3.989650	1.321070
61.H	-8.830004	-0.787976	2.699594
62.H	3.747369	5.520330	2.297097
63.H	-8.764338	-3.012595	3.608719
64.H	-2.185796	-5.036669	4.254076
65.H	-1.189612	-2.812634	4.456809
66.H	-3.954080	2.551871	4.719265
67.H	-8.645912	-1.071043	5.508955
68.H	-5.615719	-3.982320	5.382922
69.H	-7.361536	0.047855	5.923852
70.H	-4.340684	-4.849292	6.222402
71.H	-7.373305	-2.956822	6.457370
72.H	-1.572909	-4.200494	6.428018
73.H	-2.414396	2.521996	6.610309
74.H	-4.757858	2.909105	6.995551
75.H	-7.668715	-1.718979	7.701724
76.H	-5.736385	-3.713305	7.928484
77.H	-3.227978	-0.874409	7.719088
78.H	-4.127657	-2.942130	7.845383
79.H	-6.152211	0.004132	7.890563
80.H	-3.823765	0.642111	8.379085
81.H	-5.397047	-1.196993	8.973671
82.N	2.671143	-5.161611	-5.359992
83.N	4.862627	-6.438995	-4.094636
84.N	2.090949	6.657489	-3.643690
85.N	4.875250	-4.005892	-2.682871
86.N	2.989038	-6.929805	-2.061213
87.N	2.427176	8.416478	-1.447763
88.N	-0.215422	7.610959	-1.049938
89.N	3.011124	5.887969	-0.264715
90.N	-6.810738	-1.352744	4.383986
91.N	-3.690852	-3.026528	5.263506
92.N	-4.078744	0.416056	6.237245
93.N	-5.674753	-1.810817	6.937094
94.Sb	-0.050777	-0.473690	-3.444325
95.Sb	0.229610	-3.081496	-2.561439
96.Sb	-2.688454	-0.074953	-2.474587
97.Sb	-0.099130	3.155123	-2.266674
98.Sb	1.536540	0.981429	-1.677783
99.Sb	-2.434151	2.345336	-1.021382
100.Sb	-3.001156	-2.030935	-0.442499
101.Sb	-0.325425	-2.651508	0.127241
102.Sb	0.746011	-0.108111	0.814080

103.Sb	-1.516512	1.443404	1.455063
104.Sb	-3.691001	-0.276170	1.597897
105.Si	2.133741	-4.161684	-6.694497
106.Si	2.531501	5.657355	-5.011973
107.Si	-1.931726	7.720155	-1.356784
108.Si	1.690554	-7.663416	-1.141455
109.Si	5.495020	-3.138032	-1.263024
110.Si	3.353787	4.767199	1.059411
111.Si	-7.898851	-1.879766	3.130656
112.Si	-2.116244	-3.794324	5.086465
113.Si	-3.786505	2.141802	6.145205
114.U	2.896087	-4.955712	-3.149030
115.U	1.281901	6.045598	-1.651715
116.U	-4.584789	-1.085157	4.645748

Energy: −595.86282511 eV
